# Gender discrepancies in predictors for newly onset cardiovascular events and metabolic syndrome in elderly patients from rural China

**DOI:** 10.3389/fcvm.2022.995128

**Published:** 2022-11-23

**Authors:** Shasha Yu, Xiaofan Guo, GuangXiao Li, Hongmei Yang, Liqiang Zheng, Yingxian Sun

**Affiliations:** ^1^Department of Cardiology, First Hospital of China Medical University, Shenyang, China; ^2^Department of Clinical Epidemiology, Institute of Cardiovascular Diseases, First Hospital of China Medical University, Shenyang, China; ^3^Department of Clinical Epidemiology, Shengjing Hospital of China Medical University, Shenyang, China

**Keywords:** metabolic syndrome, cardiovascular events, elderly, risk factors, incidence

## Abstract

**Objective:**

The study aimed to estimate the possible predictors of cardiovascular events (CVEs) in elderly patients with metabolic syndrome (MetS) from rural China. Moreover, we also attempted to find the potential risk factors for newly diagnosed MetS.

**Methods:**

The Northeast China Rural Cardiovascular Health Study (NCRCHS) is a community-based prospective cohort study carried out in rural areas of northeast China. Approximately 1,059 elderly patients with MetS but no cardiovascular diseases and 1,565 elderly patients without MetS at baseline were enrolled in this study. They underwent a physical examination, completed a questionnaire in 2012–2013, and were followed up during 2015–2017. Cox proportional hazard analysis was conducted to assess the possible predictors of newly developed CVEs, and multivariate analysis was used to estimate the risk factors of newly diagnosed MetS.

**Results:**

The common predictors of newly developed CVEs in both men and women were family history of stroke (HR [hazard ratios] _female_: 1.696; HR _male_: 2.504) and soybean consumption (HR _female_: 0.253; HR _male_: 0.130). Moreover, minority race (HR: 0.109), systolic blood pressure (SBP) (HR: 1.021), current drinking habits (HR: 2.551), family history of hypertension (HR: 2.297), LDL-C (HR: 1.669), 5,000–20,000 CNY/year annual income (HR: 0.290), and strenuous physical activity (HR: 0.397) were predictors of CVEs only in male elderly patients. For newly diagnosed MetS, body mass index (OR _female_: 1.212; OR _male_: 1.207) and fasting blood glucose (OR _female_: 1.305; OR _male_: 1.217) were common risk factors in both genders, whereas age (OR:0.958) was a protective factor in men and > 9-h/day sleep duration (OR:0.212) was a protective factor in women. In addition, SBP (OR:1.014) ≥4 times/day, bean consumption (OR:1.955), and uric acid (OR:1.005) increased the risk of MetS in men but not in women.

**Conclusion:**

Our study identified many effective predictors of CVEs in rural elderly patients with MetS and confirmed the presence of a gender-based discrepancy. Moreover, we also identified additional risk factors, along with the traditional ones, for newly diagnosed MetS in rural elderly patients.

## Introduction

During the past decades, the rapid development of economic growth, changes in lifestyle, and longer life expectancy have led to an increase in the geriatric population worldwide. According to the Seventh National Population Census in 2020, the share of the population aged above 60 and 65 years increased by 5.44% (from 13.26% in 2010 to 18.70% in 2020) and 4.6% (from 8.9 to 13.5%) in past 10 years, respectively (1). The percentage of subjects aged 60 and 65 years and above in rural China was 23.8 and 17.7%, which is 7.99%, and 6.61% higher than those in urban areas, respectively (1). Age-related metabolic disorders, such as hypertension, diabetes, obesity, and dyslipidemia, gradually became more prevalent in this aging population. A Korean cardiometabolic syndrome survey reported an increasing case of metabolic syndrome with an increase in age, with the highest rate among subjects older than 60 (37.9%) (2). The National Survey of Health, Wellbeing, and Aging survey also showed that the prevalence of MetS was 66.0% in older women and 47.1% in older men (3–5) and is relatively more prevalent among urban than rural residents (6, 7). However, data from our study indicated that the prevalence of MetS among general rural residents was 39.0%, which was significantly higher than many other rural Chinese areas, such as Hanzhong (15.1%), Nantong (23.3%), and Xinjiang (14.43%) (8–10). The elderly population with MetS consisted of a large proportion of those in rural areas. It is well-established how metabolic disorders significantly correlate with cardiovascular events (CVEs), cancer, psoriasis, erectile dysfunction, osteoporosis, and many other non-communicable diseases (11–13). Moreover, the prognosis of dyslipidemia correlated with increased atherosclerotic extension in prediabetic and newly diagnosed patients with type 2 diabetes (T2D) (14). Studies showed how obesity has increased the risk of coronary and peripheral atherosclerotic burden (15). In addition, hyperglycemia has been reported to be associated with a higher coronary and peripheral atherosclerotic burden in patients with non-diabetes (16). In summary, MetS has led to an increased incidence of cardiovascular disease, coronary heart disease, and stroke (13, 17, 18). This observation warrants the identification of the possible predictors of cardiovascular events (CVEs) in rural elderly patients with MetS to develop effective preventive strategies. Cardiovascular diseases (CVD) have become the primary cause of mortality among elderly patients in recent years. However, the improvement in prevention, diagnosis, and treatment of cardiovascular risk factors induced a fall in CVD-mediated mortality in developed countries (19, 20). Nevertheless, the gradual prevalence of metabolic disorders such as obesity, hypertension, diabetes, and dyslipidemia in rural China synchronously increased CVD mortality (21, 22). Most previous studies to estimate the possible risk factors of MetS in rural China had cross-sectional designs, leading to less convincing conclusions. Therefore, in this study, we aimed to identify the potential risk factors of newly diagnosed MetS among rural elderly residents and determine if gender-related differences affected the prognosis.

## Methods

### Participants

The Northeast China Rural Cardiovascular Health Study (NCRCHS) is a community-based prospective cohort study carried out in the rural areas of northeast China. The specific sampling methods and the admission criteria have been described previously (23).

A multistage, stratified, and random cluster sampling design was used for our analysis. Dawa, Zhangwu, and Liaoyang were chosen in the first stage of the study from the eastern, southern, and northern regions of Liaoning Province. In the next phase, one town was selected randomly from each area (a total of 3 towns). In the third step, 8–10 rural villages were randomly chosen from each town, with the total number being 26. All eligible permanent residents (aged ≥ 35 years) were invited from each village to participate in the study. The exclusion criteria for the study included pregnant women, patients with malignant tumors, or subjects with mental disorders (23). The ethics committee approved the study of the China Medical University (Shenyang, China, AF-SDP-07-1, 0-01). At the beginning of the study, 64 subjects who had missing data or refused participation were excluded. During 2015–2017, follow-up was lost for 474 participants, resulting in 2,275 of them remaining in the final analysis. The subjects enrolled in this study are listed below.

Participants underwent a physical examination that included anthropometric parameters, blood pressure (BP), and regular blood tests during baseline (2012–2013) and follow-up (2015–2017). Besides, they also answered a questionnaire published earlier (23), providing all the relevant data, such as personal details, medical history, and socioeconomic situation. Finally, 128 elderly participants were diagnosed with new onset CVEs with MetS at baseline, and 349 elderly participants were newly diagnosed with MetS without the syndrome at baseline.

### Baseline data

History of stroke, coronary heart disease (CHD), and heart failure at baseline were labeled as self-reported and confirmed by medical records. Weight and height were measured with participants wearing lightweight clothing and no shoes. Waist circumference was measured at the umbilicus using non-elastic tape. Body mass index (BMI) was calculated as weight in kilograms divided by the square of height in meters. Blood pressure (BP) was assessed three times, with participants seated after at least 5 min of rest using a standardized automatic electronic sphygmomanometer (HEM-907; Omron, Tokyo, Japan). Fasting blood samples were collected in the morning from participants after at least a 12-h fast. Moreover, fasting plasma glucose (FPG), low-density lipoprotein cholesterol (LDL-C), high-density lipoprotein cholesterol (HDL-C), triglyceride (TG), and other routine blood biochemical indexes were enzymatically analyzed. Physical activity, which was divided into three classes, namely, low, moderate, and heavy, included occupational and leisure-time activities, as described in a previous study (21). Participants were asked to recall their food consumption details from the previous year to evaluate their dietary patterns. The questionnaire also required details about the typical weekly consumption of various foods. The stated intake was roughly measured in terms of grams per week. Vegetable consumption was assessed according to the following scale: rarely = 3, <1,000 g = 2, 1,000–2,000 g = 1, ≥2,000 g = 0. Meat consumption, including red meat, fish, and poultry, was assessed according to the following scale: rarely = 0, <250 g = 1, 250–500 g = 2, and ≥500 g = 3. Each participant received a unique diet score (meat consumption score plus vegetable consumption score, which ranges from 0 to 6). Lower diet scores suggested adherence to the Chinese diet, whereas higher ones indicated more meat intake, lower vegetable consumption, and greater adherence to a Westernized diet. The ATTICA trial used the same formulas for generating a diet (21). MetS was diagnosed following the unified criteria identified at the meeting between several major organizations in 2009 (24). The presence of any three of the following five risk factors leads to the diagnosis of metabolic syndrome: (1) elevated waist circumference (population- and country-specific definitions): ≥90 cm for men; ≥80 cm for women (Asians, Japanese, and South and Central Americans); (2) elevated triglycerides (drug treatment for elevated triglycerides is an alternate indicator): ≥150 mg/dl (1.7 mmol/L); (3) reduced HDL-C (drug treatment for reduced HDL-C is an alternate indicator): <40 mg/dl (1.0 mmol/L) in men; <50 mg/dl (1.3 mmol/L) in women; (4) elevated blood pressure (antihypertensive drug treatment in a patient with a history of hypertension is an alternate indicator): systolic ≥130 and/or diastolic ≥85 mmHg of blood pressure; and (5) elevated fasting glucose (drug treatment of elevated glucose is an alternate indicator): ≥100 mg/dl.

### Follow-up

The median follow-up for the study was 4.66 years. An incident CVE was defined as a composite of a new-onset stroke or CHD during the follow-up period. The specific incidences of stroke and CHD were also determined for our analyses. All available clinical information was collected for all the participants reporting possible diagnoses, including medical records and death certificates. All materials were independently reviewed and adjudicated by the end-point assessment committee. Stroke was defined as rapidly developing signs of focal or global disturbance of cerebral function, lasting more than 24 h (unless interrupted by surgery or death), with no apparent non-vascular causes, according to the WHO Multinational Monitoring of Trends and Determinants in Cardiovascular Disease (MONICA) criteria (25, 26). Hemorrhagic stroke was defined as stroke cases diagnosed with subarachnoid hemorrhage or intracerebral hemorrhage. Ischemic stroke was defined as stroke cases diagnosed with thrombosis or embolism. Transient ischemic attack and chronic cerebral vascular disease were excluded from our study. CHD included cases of diagnosed hospitalized angina, hospitalized myocardial infarction, CHD death, or any revascularization procedure (27).

### Statistical analysis

Statistical analyses were performed for all the variables, including the continuous (reported as mean values and standard deviations) and categorical (reported as numbers and percentages) ones. Significant differences between the various groups were evaluated using the *t*-test, ANOVA, the non-parametic test, or the χ*2-*test, as appropriate. The hazard ratios of CVEs for each unit increment of metabolic parameters and other possible confounders were calculated by Cox's proportional-hazard model. Logistic regression analyses were used to estimate odds ratios (ORs) and 95% confidence intervals (CIs) for the potential risk factors of newly diagnosed MetS after adjusting for possible confounders. All the statistical analyses were performed using SPSS version 20.0 software (SPSS Inc., Chicago, Illinois, USA), and *p*-values < 0.05 were considered statistically significant.

## Results

### Gender-wise evaluation of the baseline characteristics of elderly patients with MetS

Demographic data for 1,059 elderly subjects with baseline MetS in the period 2012–2013 are presented in Table 1. Among elderly patients with MetS, women had a significantly higher rate of primary school education or even lower educational status (85.5 vs. 61.3%), did light physical activity (68.1 vs. 49.7%), and had shorter sleep duration (≤7 h/day: 61.5 vs. 52.1%) compared to their male counterparts, were more likely to use glucose-lowering (9.9 vs. 4.1%) and antihypertensive medications (41.6 vs. 34.7%), and were unlikely to consume soybean and soybean products (42.6 vs. 28.5). On the contrary, male elderly patients with MetS showed a higher diet score (51.1 vs.32.3%), were more likely to have alcohol consumption habits (34.5 vs. 3.0%), and were smokers (42.8 vs. 20.2%). Considering biochemical parameters, male elderly patients with MetS had significantly higher SBP, DBP, and uric acid but lower LDL-C than female elderly patients.

**Table 1:** Baseline characteristics of rural northeast Chinese elderly patients with metabolic syndrome.

**Variables**	**Total (*n =* 1059)**	**Men (*n =* 362)**	**Women (*n =* 697)**	***P*-value**
**Age (years)**	66.59 ± 5.48	66.40 ± 5.50	66.68 ± 5.47	0.308
**Ethnicity**				0.394
Han	1014 (95.8)	348 (96.1)	666 (95.6)	
Others^a^	45 (4.2)	14 (3.9)	31 (4.4)	
**Education status**				<0.001
Primary school or below	818 (77.2)	222 (61.3)	596 (85.5)	
Middle school	198 (18.7)	114 (31.5)	84 (12.1)	
High school or above	43 (4.1)	26 (7.2)	17 (2.4)	
**Physical activity**				<0.001
Light	645 (61.8)	178 (49.7)	467 (68.1)	
Moderate	173 (16.6)	66 (18.4)	107 (15.6)	
Severe	226 (21.6)	114 (31.8)	112 (16.3)	
**Annual income (CNY/year)**				0.070
≤5,000	229 (21.6)	75 (20.7)	154 (22.1)	
5,000–20,000	589 (55.7)	190 (52.5)	399 (57.3)	
>20,000	240 (22.7)	97 (26.8)	143 (20.5)	
**Sleep duration**, ***n*** **(%)**				0.006
≤7 h/day	615 (58.3)	188 (52.1)	427 (61.5)	
7–8 h/day	227 (21.5)	80 (22.2)	147 (21.2)	
8–9 h/day	138 (13.1)	59 (16.3)	79 (11.4)	
>9 h/day	75 (7.1)	34 (9.4)	41 (5.9)	
**Soybean and soybean product consumption**				<0.001
Rare consumption	398 (37.7)	103 (28.5)	295 (42.6)	
2–3 times/week	518 (49.1)	196 (54.1)	322 (46.5)	
≥4 time/week	139 (13.2)	64 (17.4)	76 (11.0)	
**Current smoking status (Yes)**	296 (28.0)	155 (42.8)	141 (20.2)	<0.001
**Current drinking status (Yes)**	146 (13.8)	125 (34.5)	21 (3.0)	<0.001
**Diet score (≥3)**	410 (38.8)	185 (51.1)	225 (32.3)	<0.001
**Lipid-lowering Medication** (Yes)	63 (5.9)	19 (5.2)	44 (6.3)	0.292
**Glucose-lowering Medication** (Yes)	84 (7.9)	15 (4.1)	69 (9.9)	<0.001
**Antihypertensive Medication** (Yes)	332 (39.1)	105 (34.7)	227 (41.6)	0.028
**Family history of hypertension**	232 (21.9)	82 (22.7)	150 (21.2)	0.364
**Family history of CHD**	126 (11.9)	37 (10.2)	89 (12.8)	0.132
**Family history of Stroke**	196 (18.5)	66 (18.2)	130 (18.7)	0.469
**SBP (mmHg)**	157.43 ± 23.34	159.01 ± 22.37	156.61 ± 23.80	<0.001
**DBP (mmHg)**	84.37 ± 11.50	87.63 ± 10.78	82.68 ± 11.50	<0.001
**BMI (kg/m** ^**2**^ **)**	26.17 ± 3.36	26.42 ± 2.79	26.04 ± 3.61	0.076
**LDL-C (mmol/L)**	3.28 ± 0.89	3.09 ± 0.86	3.38 ± 0.90	<0.001
**Uric acid (mmol/L)**	305.60 ± 86.65	351.98 ± 90.02	281.51 ± 74.24	<0.001
**FPG (mmol/L)**	6.60 ± 2.22	6.60 ± 2.28	6.60 ± 2.18	0.962

Data are expressed as the mean ± SD or as *n* (%). BMI, body mass index; WC, waist circumference; CNY, China yuan (1 CNY = 0.161 USD); SBP, systolic blood pressure; DBP, diastolic blood pressure; LDL-C, low-density lipoprotein cholesterol; FPG, fasting plasma glucose.

a Including some ethnic minorities in China, such as Mongol and Manchu.

### Gender-wise changes in biochemical parameters from baseline to follow-up in elderly patients with or without MetS

**Figure 1:**
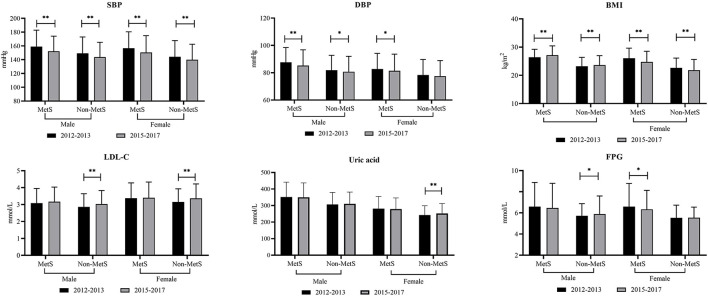
Changes in different metabolic parameters from 2012–2013 to 2015–2017 among rural elderly patients with or without metabolic syndrome. * means *P* < 0.05; ** means *P* < 0.001; ns means without significant difference; MetS, metabolic syndrome; SBP, systolic blood pressure; DBP, diastolic blood pressure; WC, waist circumference; BMI, body mass index; FPG, fasting plasma glucose; TG, triglycerides; LDL-C, low density lipid cholesterol; HDL-C, high density lipid cholesterol.

Figure 1 shows that 1,059 elderly patients were diagnosed with MetS at baseline. We aimed to evaluate the past changes in metabolic parameters such as SBP, DBP, WC, BMI, and biochemical parameters among the elderly with or without MetS (Figure 1). From 2012–2013 to 2015–2017, male participants with MetShad significantly decreased SBP and DBP but increased BMI. In 2012–2013, female participants with MetS had relatively higher values of SBP, DBP, BMI, and FPG. For subjects free of MetS, male participants had higher SBP and DBP but lower BMI, LDL, and FPG. Female participants free of MetS at baseline had significantly higher values of SBP and BMI, whereas LDL-C and uric acid were relatively lower.

### The possible predictors of CVEs among elderly patients with MetS at baseline

We conducted Cox's proportional-hazard analysis to determine the possible predictors of CVEs among elderly patients with MetS and estimated the possible presence of a gender discrepancy. Data in Table 2 show that a family history of stroke (HR _formale_: 2.504; HR _forfemale_: 1.696) and 2–3 times/week soybean and soybean product consumption (HR _formale_: 0.130; HR _forfemale_: 0.253) were conjunct predictors for CVEs in both male and female participants. In addition, ancestry of Han race [HR (95%CI): 0.109 (0.022,0.535)], family history of hypertension [HR (95%CI): 2.359 (1.032,5.391)], SBP [HR (95%CI): 1.021 (1.001,1.040)], LDL-C [HR (95%CI): 1.681 (1.130,2.502)], uric acid [HR (95%CI): 0.995 (0.990,0.999)], hemoglobin level [HR (95%CI): 0.972 (0.947,0.997)], 5,000–20,000 CNY/year annual income [HR (95%CI): 0.285 (0.124,0.652)], alcohol consumption habits [HR (95%CI): 2.551 (1.159,5.611)], and heavy physical activity [HR (95%CI): 0.393 (0.155,0.995)] were effective predictors of CVEs among male participants but not in female participants.

**Table 2:** Cox proportional hazard model: Hazard ratio of newly onset cardiovascular events among elderly patients with MetS at baseline (*n* = 1,059).

	**Male**				**Female**			
**Variable**	**Hazard ratio**	**HR lower CL**	**HR upper CL**	***P*-value**	**Hazard ratio**	**HR lower CL**	**HR upper CL**	***P*-value**
**Age at entry** (per 1 year)	0.983	0.919	1.052	**0.495**	**1.073**	**1.016**	**1.132**	**0.005**
**Race** (minority as refer)	**0.109**	**0.022**	**0.542**	**0.005**	0.760	0.232	2.485	0.804
**Family history of CHD** (no as refer)	1.007	0.263	3.858	0.972	1.067	0.575	1.845	0.406
**Family history of Stroke** (no as refer)	**2.504**	**1.141**	**5.498**	**0.048**	**1.696**	**1.023**	**2.645**	**0.043**
**Family history of Hypertension** (no as refer)	**2.297**	**0.997**	**5.293**	**0.009**	1.430	0.753	2.717	0.365
**SBP** (per 1 mmHg)	**1.021**	**1.001**	**1.040**	**0.050**	1.014	0.999	1.029	0.298
**DBP** (per 1 mmHg)	1.021	0.982	1.063	0.321	0.997	0.972	1.023	0.633
**BMI** (per 1kg/m^2^)	1.129	0.986	1.294	0.051	0.967	0.893	1.047	0.676
**FPG** (per 1 mmol/L)	0.790	0.612	1.019	0.065	1.036	0.924	1.162	0.838
**LDL-C** (per 1 mmol/L)	**1.669**	**1.119**	**2.488**	**0.009**	1.089	0.794	1.492	0.637
**Uric acid** (per 1 mmol/L)	**0.995**	**0.990**	**0.999**	**0.040**	0.998	0.995	1.002	0.198
**Hemoglobin** (g/L)	**0.972**	**0.947**	**0.997**	**0.048**	0.998	0.977	1.020	0.587
**Sleep duration** (≤7 h/day as refer)								
7–8 h/day	1.628	0.667	3.971	0.568	1.164	0.574	2.360	0.781
8–9 h/day	0.627	0.186	2.127	0.309	1.604	0.693	3.712	0.228
>9 h/day	1.818	0.611	5.413	0.346	2.071	0.728	5.886	0.349
**Education status** (Primary school or below as refer)								
Middle school	0.511	0.236	1.106	0.102	0.918	0.363	2.320	0.473
High school or above	0.367	0.141	1.801	0.091	3.898	0.949	16.021	0.109
**Annual income (CNY/year)** (≤ 5000 as refer)								
5,000–20,000	**0.290**	**0.126**	**0.669**	**0.016**	1.040	0.526	2.053	0.932
>20,000	0.367	0.131	1.029	0.078	1.042	0.438	2.482	0.842
**Physical activity** (Light as refer)								
Moderate	0.740	0.285	1.921	0.273	0.968	0.461	2.036	0.971
Heavy	**0.397**	**0.157**	**0.997**	**0.016**	0.678	0.246	1.868	0.724
**Soybean and soybean product consumption**								
2–3 times/week	0.907	0.432	1.907	0.890	0.552	0.305	1.001	0.052
≥4 time/week	**0.130**	**0.030**	**0.555**	**0.014**	**0.253**	**0.075**	**0.854**	**0.035**
**Current smoking** (no as refer)	1.622	0.801	3.283	0.091	1.292	0.668	2.499	0.353
**Current drinking** (no as refer)	**2.551**	**1.159**	**5.611**	**0.033**	0.548	0.064	4.657	0.954
**Lipid lowering Medication** (Yes as refer)	0.664	0.602	5.934	0.624	0.742	0.234	2.351	0.365
**Glucose lowering Medication** (Yes as refer)	0.517	0.090	3.359	0.445	1.661	0.694	3.978	0.182
**Antihypertensive Medication** (Yes as refer)	1.134	0.547	2.352	0.881	1.634	0.893	2.989	0.120
**Diet score (≥3)**	1.148	0.578	2.278	0.521	0.930	0.504	1.716	0.712

SBP, systolic blood pressure; DBP, diastolic blood pressure; LDL-C, low-density lipoprotein cholesterol; FBG, fasting plasma glucose. The bold value indicates the value of *P* < 0.05.

### Gender-wise baseline characteristics of elderly patients without MetS

Demographic data for 1,216 elderly patients without MetS at baseline in 2012–2013 are presented in Table 3. Male participants without MetS at baseline were relatively older, had a higher proportion of Han ethnicity (96.4 vs. 93.9%, *P* = 0.037), were smokers (52.1 vs. 27.4%, *P* < 0.001), consumed alcohol (41.0 vs. 5.4%, *P* < 0.001), and had an elevated diet score (49.2 vs. 33.4%, *P* < 0.001) but had a lower rate of primary school or even lower educational status (61.3 vs. 85.5%, *P* < 0.001), did light physical activity (49.7 vs. 60.1%, *P* < 0.001), had ≤7 h/day sleep duration (49.1 vs. 62.9%, *P* < 0.001), rarely consumed soybean (35.2 vs. 43.2%, *P* = 0.020), and had a family history of CHD (7.5 vs. 13.9%, *P* < 0.001). The SBP, DBP, BMI, uric acid, and FPG levels were significantly higher in male participants, whereas LDL-C was lower in male participants.

**Table 3:** Baseline characteristics of rural northeast Chinese elderly patients without metabolic syndrome.

**Variables**	**Total (*n =* 1216)**	**Men (*n =* 770)**	**Women (*n =* 446)**	***P*-value**
**Age (years)**	66.57 ± 5.54	66.99 ± 5.74	65.85 ± 5.10	<0.001
**Ethnicity**				0.037
Han	1161 (95.5)	742 (96.4)	419 (93.9)	
Others ^a^	55 (4.5)	28 (3.6)	27 (6.1)	
**Education status**				<0.001
Primary school or below	818 (77.2)	222 (61.3)	596 (85.5)	
Middle school	198 (18.7)	114 (31.5)	84 (12.1)	
High school or above	43 (4.1)	26 (7.2)	17 (2.4)	
**Physical activity**				<0.001
Light	644 (53.8)	379 (49.7)	265 (60.1)	
Moderate	197 (16.4)	121 (15.9)	76 (17.2)	
Severe	362 (30.1)	262 (34.4)	100 (22.7)	
**Annual income (CNY/year)**				0.718
≤5,000	280 (23.1)	177 (23.0)	103 (23.1)	
5,000–20,000	709 (58.5)	444 (57.8)	265 (59.8)	
>20,000	224 (18.5)	147 (19.1)	77 (17.3)	
**Sleep duration**, ***n*** **(%)**				<0.001
≤7 h/day	657 (54.2)	377 (49.1)	280 (62.9)	
7–8 h/day	307 (25.3)	206 (26.8)	101 (22.7)	
8–9 h/day	166 (13.7)	120 (15.6)	46 (10.3)	
>9 h/day	83 (6.8)	65 (8.5)	46 (4.0)	
**Soybean and soybean product consumption**				0.020
Rare consumption	463 (38.2)	271 (35.2)	192 (43.2)	
2–3 times/week	613 (50.5)	409 (53.2)	204 (45.9)	
≥4 time/week	137 (11.3)	89 (11.6)	48 (10.8)	
**Diet score (≥3)**	528 (43.4)	379 (49.2)	149 (33.4)	<0.001
**Current smoking status (Yes)**	523 (43.0)	401 (52.1)	122 (27.4)	<0.001
**Current drinking status (Yes)**	340 (28.0)	316 (41.0)	24 (5.4)	<0.001
**Family history of hypertension**	195 (16.0)	114 (14.8)	81 (18.2)	0.073
**Family history of CHD**	120 (9.9)	58 (7.5)	62 (13.9)	<0.001
**Family history of Stroke**	197 (16.2)	120 (15.6)	77 (17.3)	0.246
**SBP (mmHg)**	147.47 ± 23.68	149.39 ± 23.53	144.14 ± 23.60	<0.001
**DBP (mmHg)**	80.54 ± 11.19	81.81 ± 10.93	78.36 ± 11.32	<0.001
**BMI (kg/m** ^**2**^ **)**	23.01 ± 3.28	23.22 ± 3.14	22.65 ± 3.47	0.004
**LDL-C (mmol/L)**	2.96 ± 0.79	2.86 ± 0.78	3.15 ± 0.78	<0.001
**Uric acid (mmol/L)**	283.06 ± 74.32	306.82 ± 72.89	242.11 ± 56.95	<0.001
**Hemoglobin (g/L)**	139.04 ± 17.35	143.93 ± 17.41	130.55 ± 13.59	<0.001
**FPG (mmol/L)**	5.65 ± 1.17	5.73 ± 1.14	5.53 ± 1.20	0.004

Data are expressed as the mean ± SD or as n (%). BMI, body mass index; WC, waist circumference; CNY, China yuan (1 CNY = 0.161 USD); SBP, systolic blood pressure; DBP, diastolic blood pressure; LDL-C, low-density lipoprotein cholesterol; FPG, fasting plasma glucose.

a Including some ethnic minorities in China, such as Mongol and Manchu.

### Cumulative incidence of newly diagnosed MetS among elderly patients

**Figure 2:**
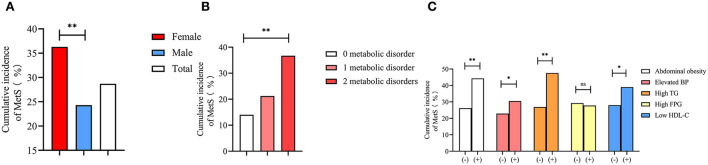
Cumulative incidence of metabolic syndrome among gender **(A)**, different numbers of metabolic disorders at baseline **(B)**, and different metabolic disorders **(C)**. * means *P* < 0.05; ** means *P* < 0.001; ns means without significant difference; BP, blood pressure; TG, triglyceride; FPG, fasting plasma glucose; HDL-C, high density lipid cholesterol.

In the present study, 1,216 elderly patients did not have MetS at baseline. The cumulative incidence of newly diagnosed MetS among these 1,216 patients during the 4.66 years of follow-up is shown in Figure 2, which showed a significantly higher rate of MetS in women than in men (36.3 vs. 24.4%, *P* < 0.001) (Figure 2A). Moreover, increased incidences of metabolic disorders led to an increase in the number of newly diagnosed MetS cases (14.0, 21.2, and 36.7%, *P* for trend < 0.001) (Figure 2B). The presence of metabolic disorders at baseline in the elderly patients predisposed them toward developing MetS during the follow-up (Figure 2C). Elderly patients with abdominal obesity (44.3 vs. 26.2%, *P* < 0.001), elevated BP (30.5 vs. 22.9%, *P* = 0.008), high TG (47.6 vs. 26.9%, *P* < 0.001), and low HDL-C (39.0 vs. 28.0%, *P* < 0.001) had a significantly higher rate of newly diagnosed MetS compared with subjects free of these symptoms. The elderly patients with high TG had the highest rate of newly diagnosed MetS (47.6%).

### Possible associated factors of newly diagnosed MetS

We conducted a multivariable analysis to identify the possible risk factors that might affect the incidence of MetS (Table 4). Between the two genders, data showed that a 1 unit increase in BMI (OR _formale_: 1.212; OR _forfemale_: 1.207) and FPG (OR _formale_: 1.217; OR _forfemale_: 1.305) led to an increased risk of newly diagnosed MetS. In addition, age (OR: 0.958) was identified as a protective factor for newly diagnosed MetS among men. LDL-C (OR: 1.771) and current smoking habits (OR: 1.728) were also identified as risk factors for newly diagnosed MetS among female elderly patients but not among male elderly patients. Sleep duration of > 9 h/day (OR: 0.212) was associated with a lower risk of newly diagnosed MetS among female elderly patients.

**Table 4:** Multivariable analysis of possible associations between risk factors and cumulative incidence of MetS among elderly patients without MetS at baseline (*n* = 1,216).

	**Male**				**Female**			
**Variable**	**Odd ratio**	**95%CI (lower)**	**95%CI (upper)**	***P*-value**	**Odd ratio**	**95%CI (lower)**	**95%CI (upper)**	***P*-value**
**Age at entry** (per 1 year)	**0.958**	**0.922**	**0.996**	**0.025**	1.015	0.964	1.070	0.555
**Race** (minority as refer)	1.740	0.536	5.648	0.367	0.531	0.208	1.356	0.234
**SBP** (per 1 mmHg)	**1.014**	**1.003**	**1.025**	**0.005**	1.009	0.997	1.022	0.142
**DBP** (per 1 mmHg)	0.999	0.977	1.022	0.936	0.992	0.966	1.019	0.570
**BMI** (per 1kg/m^2^)	**1.212**	**1.126**	**1.304**	**0.010**	**1.207**	**1.116**	**1.306**	**0.034**
**FPG** (per 1 mmol/L)	**1.217**	**1.046**	**1.415**	**<0.001**	**1.305**	**1.015**	**1.678**	**<0.001**
**LDL-C** (per 1 mmol/L)	1.215	0.961	1.538	0.097	**1.771**	**1.305**	**2.404**	**<0.001**
**Uric acid (mmol/L)**	**1.005**	**1.002**	**1.007**	**0.001**	1.003	0.999	1.007	0.124
**Hemoglobin** (g/L)	1.005	0.994	1.016	0.454	1.005	0.987	1.022	0.608
**Sleep duration** (≤7 h/day)								
7–8 h/day	0.960	0.611	1.509	0.979	0.876	0.508	1.517	0.569
8–9 h/day	0.674	0.382	1.189	0.179	0.490	0.214	1.120	0.088
>9 h/day	1.558	0.828	2.932	0.158	**0.212**	**0.050**	**0.890**	**0.031**
**Education status** (Primary school or below as refer)								
Middle school	1.254	0.830	1.893	0.267	1.298	0.675	2.496	0.471
High school or above	1.233	0.528	2.877	0.556	0.218	0.021	2.301	0.220
**Annual income (CNY/year)** (≤ 5,000 as refer)								
5,000–20,000	0.888	0.557	1.417	0.626	0.677	0.382	1.198	0.168
>20,000	0.694	0.375	1.283	0.168	0.880	0.411	1.882	0.627
**Physical activity** (Light as refer)								
Moderate	1.138	0.858	1.969	0.599	0.711	0.374	1.354	0.291
Heavy	1.159	0.753	1.785	0.419	1.215	0.674	2.190	0.452
**Soybean and soybean product consumption**								
2–3 times/week	1.051	0.695	1.590	0.978	1.162	0.708	1.909	0.610
≥ 4 time/week	1.352	0.629	3.574	0.056	1.273	0.583	2.783	0.565
**Diet score (≥3)**	0.743	0.511	1.082	0.154	0.830	0.502	1.370	0.898
**Family history of hypertension** (no family history of hypertension group as reference group)	0.780	0.460	1.323	0.360	1.122	0.616	2.044	0.678
**Family history of CHD** (no family history of CHD group as reference group)	1.290	0.658	2.529	0.498	0.716	0.362	2.478	0.385
**Family history of Stroke** (no family history of stroke group as reference group)	1.276	0.764	2.131	0.257	1.334	0.729	2.044	0.324
**Current smoking** (no current smoking group as reference group)	1.337	0.891	2.006	0.120	**1.728**	**1.000**	**2.986**	**0.042**
**Current drinking** (no current drinking group as reference group)	0.781	0.524	1.165	0.206	0.571	0.191	1.704	0.375

SBP, systolic blood pressure; DBP, diastolic blood pressure; LDL-C, low-density lipoprotein cholesterol; FBG, fasting plasma glucose. The bold value indicates the value of *P* < 0.05.

## Discussion

The present study identified an increase in SBP by 1 mmHg, a family history of stroke, a soybean and soybean product consumption rate of 2–3 times/week, and alcohol consumption habits as crucial predictors of CVEs in both genders with MetS. In addition, Han Chinese's race descendancy, family history of hypertension, high levels of LDL-C, uric acid, and hemoglobin, an income range of 5,000–20,000 CNY/year, and heavy physical activity were specific predictors of CVEs among male elderly patients but not among female patients with MetS at baseline. BMI and FPG were risk factors for both genders, considering the associated factors of newly diagnosed MetS. Our analyses identified >9 h/day of sleep as a protective and LDL-C as a risk factor for newly diagnosed MetS among female elderly patients. In contrast, age and SBP were associated factors in elderly male patients but not in female patients.

One study including 6,828 adult patients with MetS concluded that factors such as the male gender, age, cigarette smoking, meat/food intake, dietary pattern, and metabolic components at the baseline significantly positively affected the risk of developing MetS (28). Hence, they suggested abstaining from cigarette smoking and dietary pattern modification as the major prevention strategies for controlling MetS. Another study showed that an intermediate level of physical activity seemed to be a protective factor for MetS (29), suggesting a need for MetS prevention strategies that promote physical activity. However, a study specifically comprising elderly patients found no difference in physical activity between older adults with MetS and those without this diagnosis (30). We corroborated this result by finding no statistically significant relationship between physical activity and newly diagnosed MetS among rural elderly patients. A possible reason behind such an observation might be the relatively low rate of moderate and heavy physical activity among older subjects due to aging. In our study, BMI and FPG at baseline were identified as common risk factors for newly diagnosed MetS in both genders, which is consistent with observations already corroborated by previous studies. SoMi Park and colleagues reported that the risk groups of metabolic indicators all showed statistically significant differences according to BMI. The percentage of subjects with abnormal findings for all indicators significantly increased with an increase in BMI (31). Moreover, female elderly patients possessed a relatively higher risk of developing MetS than male elderly patients, warranting more attention except for the common risk factors of newly diagnosed MetS in both genders. LDL-C and smoking habits were also risk factors for MetS in female elderly patients. Cigarette smoking significantly positively affects the risk of developing MetS (28). As previously emphasized, elderly patients must pay attention to their blood lipid profile, glucose, and blood pressure and monitor them frequently to discover abnormal changes as early as possible. In recent years, cumulative evidence emphasized the beneficial effects of a decrease in the levels of lipid, glucose, and antihypertensive medication on cardiovascular diseases (32–34). However, data from the DYSIS-China study reported that attaining the goals for lowering both LDL-C and non-HDL-C occurred less frequently in MetS Chinese than in those without MetS, which underscored the necessity of adding recommendations about strategies for controlling multiple risk factors to decrease the risk related to dyslipidemia in MetS subjects in future guidelines (35). In our study, >9 h/day of sleep duration was associated with a lower risk of MetS in female elderly patients. A systemic review claimed that shorter sleep durations were significantly associated with a higher prevalence of MetS (OR = 1.11, 95% CI = 1.05–1.18) and incident MetS (RR = 1.28, 95% CI = 1.07–1.53) in cross-sectional and longitudinal studies, respectively. However, long sleep duration was significantly associated with an increased prevalence of MetS in cross-sectional studies (OR = 1.14, 95% CI = 1.05–1.23) but not with the incidences of MetS (RR = 1.16, 95% CI = 0.95–1.41) in longitudinal studies (36). Furthermore, other reviews highlighted a U-shaped association between sleep duration and MetS. It has been reported that short and long sleep durations are associated with a higher risk of MetS in women (37). Future studies are needed to shed light on the underlying mechanisms associated with sleep duration and MetS and examine if normalizing sleep duration reduces MetS risk in the general population.

Increasing evidence regarding the association between MetS and mortality confirmed the association between MetS and a higher risk of all-cause mortality and CVD mortality among older subjects (38). Besides, a growing body of suggests that elderly patients with MetS present a higher incidence of CVEs than those without the condition (39, 40), warranting the need to identify and manage individual risk factors for CVEs among elderly patients with MetS. Many strategies, including lifestyle changes, might effectively decrease CVEs among subjects with metabolic syndrome. Clinical trials demonstrated that intensive lifestyle modifications, such as increased physical activity and dietary changes, can reduce the risk of CVEs (41). However, in our study, the beneficial effect of physical activity on CVEs was only significant in elderly male patients with MetS, despite multiple previous studies identifying the protective effect of physical activity on CVEs (42–44). The Mediterranean-style diet, as a recommended dietary pattern, was associated with a reduced risk of CVEs (45), as patients with MetS following this diet significantly reduced body weight, WC, BP, plasma glucose, total cholesterol, and TG, as well as improved HDL-C concentration (46). The meta-analysis also confirmed that increased vegetable and fruit intake was positively correlated with a reduced risk of stroke and CHD (47). In addition, data in our analysis established soybean and soybean product consumption as beneficial predictors of CVEs in both genders. Barańska et al. found that soy and its isoflavones can effectively correct changes in the lipid metabolism in postmenopausal women and may favorably influence the prevention of CVEs (48). The second effective strategy included abstaining from drinking. In our study, alcohol consumption was a common predictor of CVEs in both genders, confirming the necessity to quit drinking alcohol in older stages of life. In the case of elderly male patients, being descendants of the Han race was a protective predictor of CVEs, coinciding with many previous studies. One review suggests that cardiovascular complications are more influenced by genetics and culture than diabetes mellitus and hypertension (49). Minority races in China prefer consuming meat because of weight problems and dyslipidemia, which presents a higher risk of developing CVEs. Moreover, a relatively higher annual income was associated with a lower risk of developing CVEs in elderly male patients with MetS. Shin et al. reported that low income and uncontrolled blood pressure are associated with increased all-cause and cardiovascular mortality and cardiovascular events in patients with hypertension (50). Similarly, diabetes combined with low income was associated with substantially elevated mortality risk, myocardial infarction, and ischemic stroke among primary care patients with hypertension (51). These findings collectively indicated that income is an essential aspect of social determinants of health that impacts cardiovascular outcomes in the care of MetS.

The strengths of this study include a prospective follow-up design, a defined length of follow-ups, and validated methods for determining cardiovascular events. Our study identified novel associated risk factors and predictors of newly diagnosed MetS and CVEs among elderly patients with MetS at baseline. Next, we aimed to determine effective strategies to decrease the incidence of MetS and CVEs among rural elderly patients. As shown in our study, in addition to the traditional risk factors such as dyslipidemia, hyperglycemia, hyperuricemia, hypertension, and obesity, we found soybean consumption to have a potential beneficial effect on preventing MetS and CVEs. Future studies should identify the amounts of soybean products sufficient to be given beneficially without causing MetS and CVEs.

There were some limitations in the present study. First, we did not evaluate the possible effect of diet on MetS and CVEs. Second, there were some participant losses due to their failure to show up during the follow-up, which might cause bias in the predictive effect of MetS on CHD, stroke, or CVD. Third, HDL-C, LDL-C, triglyceride, and fasting plasma glucose were measured only one time during the baseline and follow-up examination, which might be imprecise and have resulted in random errors. Third, the diet just included meat and vegetable consumption, and the diet score was calculated to represent the diet pattern. There were many other components, such as dairy products and fruits, that were not assessed in our study. Finally, the results of this study cannot be generalized to other populations.

## Conclusion

MetS cases were prevalent among the rural Chinese elderly patients, with the cumulative incidence being even higher than that in many developed or urban areas in China. Except for traditional risk factors such as BMI, FPG, LDL-C, SBP, and current smoking habits, sleep duration of >9 h/day and age was a protective factors for newly diagnosed MetS. Moreover, a history of stroke, 2–3 times/week of soybean and soybean product consumption, and alcohol consumption were good predictors of MetS in both genders in the elderly population. Race, LDL-C, uric acid, hemoglobin, 5,000–20,000 annual income, and heavy physical activity were also predictors of CVEs in male elderly patients.

## Data availability statement

The raw data supporting the conclusions of this article will be made available by the authors, without undue reservation.

## Ethics statement

The studies involving human participants were reviewed and approved by the Ethics Committee of China Medical University (Shenyang, China AF-SDP-07-1, 0-01). The patients/participants provided their written informed consent to participate in this study.

## Author contributions

SY contributed to the data collection, analysis, and interpretation. XG and HY contributed to data collection. GL and SY contributed to the data analysis. YS contributed to the study's conception and design. All authors read and approved the final version of the manuscript.

## Funding

This study was supported by grants from the National Key Research and Development Program from the Ministry of Science and Technology of China (Project Grant # 2018 YFC 1312400 and Sub-project Grant # 2018 YFC 1312403) and the ‘China Medical University Youth Backbone Program’ project funds (Grant No. QGZ2018037).

## Conflict of interest

The authors declare that the research was conducted in the absence of any commercial or financial relationships that could be construed as a potential conflict of interest.

## Publisher's note

All claims expressed in this article are solely those of the authors and do not necessarily represent those of their affiliated organizations, or those of the publisher, the editors and the reviewers. Any product that may be evaluated in this article, or claim that may be made by its manufacturer, is not guaranteed or endorsed by the publisher.
